# Occlusion of dopamine-dependent synaptic plasticity in the prefrontal cortex mediates the expression of depressive-like behavior and is modulated by ketamine

**DOI:** 10.1038/s41598-022-14694-w

**Published:** 2022-06-30

**Authors:** Jacopo Lamanna, Francesco Isotti, Mattia Ferro, Sara Spadini, Gabriella Racchetti, Laura Musazzi, Antonio Malgaroli

**Affiliations:** 1 Center for Behavioral Neuroscience and Communication (BNC), Vita-Salute San Raffaele University, Via Olgettina 58, 20132 Milan, Italy; 2 Faculty of Psychology, Vita-Salute San Raffaele University, 20132 Milan, Italy; 3 Department of Psychology, Sigmund Freud University, 20143 Milan, Italy; 4 Division of Neuroscience, Scientific Institute Ospedale San Raffaele, 20132 Milan, Italy; 5 School of Medicine and Surgery, University of Milano-Bicocca, 20900 Monza, Italy

**Keywords:** Depression, Neurophysiology, Neuroscience, Stress and resilience, Synaptic plasticity, Synaptic transmission

## Abstract

Unpredictable chronic mild stress (CMS) is among the most popular protocols used to induce depressive-like behaviors such as anhedonia in rats. Differences in CMS protocols often result in variable degree of vulnerability, and the mechanisms behind stress resilience are of great interest in neuroscience due to their involvement in the development of psychiatric disorders, including major depressive disorder. Expression of depressive-like behaviors is likely driven by long-term alterations in the corticolimbic system and by downregulation of dopamine (DA) signaling. Although we have a deep knowledge about the dynamics of tonic and phasic DA release in encoding incentive salience and in response to acute/chronic stress, its modulatory action on cortical synaptic plasticity and the following implications on animal behavior remain elusive. Here, we show that the expression of DA-dependent synaptic plasticity in the medial prefrontal cortex (mPFC) is occluded in rats vulnerable to CMS, likely reflecting differential expression of AMPA receptors. Interestingly, such difference is not observed when rats are acutely treated with sub-anesthetic ketamine, possibly through the recruitment of dopaminergic nuclei such as the ventral tegmental area. In addition, by applying the synaptic activity sensor SynaptoZip in vivo, we found that chronic stress unbalances the synaptic drive from the infralimbic and prelimbic subregions of the mPFC toward the basolateral amygdala, and that this effect is counteracted by ketamine. Our results provide novel insights into the neurophysiological mechanisms behind the expression of vulnerability to stress, as well as behind the antidepressant action of ketamine.

## Introduction

The mechanisms behind stress vulnerability and resilience are of great interest in neuroscience due to their involvement in the development of psychiatric disorders, including major depressive disorder^1–3^. Unpredictable chronic mild stress (CMS) is among the most popular protocols used to induce depressive-like behaviors such as anhedonia in rats^4^. From a translational point of view, it is important to notice that, based on individual response to stress, the exposure to CMS can result in variable degree of vulnerability, so that CMS animals not displaying anhedonia can be behaviorally defined as stress resilient, compared to vulnerable ones. Chronic stress is known to induce long-lasting, circuit-specific changes on cortico-limbic structures^5^, including the prefrontal cortex (PFC), amygdala, hippocampus, *nucleus accumbens* (NAc) and ventral tegmental area (VTA). In this context, an open question is whether functional synaptic plasticity, which is affected by chronic stress, also plays a role in the expression of depressive-like behaviors.

The dopamine (DA) system is a major actor in the development of stress-related depressive phenotypes^6^. In fact, changes in the dynamics of tonic and phasic DA release by mesolimbic regions have been found in response to both acute and chronic stressors^7,8^, and a down-regulation of the DA system has been linked to depressive-like states^6^, in line with the central role of DA in encoding incentive salience and promoting motivation^9^. Importantly, DA plays a role in modulating cortical synaptic plasticity, including long-term potentiation (LTP) and long-term depression (LTD)^10–13^, as well as spike timing dependent plasticity (STDP)^14–16^ especially on local PFC circuitry (see^17^ for review). These effects are particularly relevant in the context of PFC microcircuits, which involve different neuronal phenotypes and interconnect PFC subregions and layers^18^.

Although the mechanisms behind the induction and expression of these forms of synaptic plasticity within PFC have been deeply characterized in vitro, their implications on animal behavior remain elusive^11,12,17,19^. Nevertheless, DA-dependent PFC synaptic plasticity is likely central to the development of depressive-like behaviors because DA could affect the long-term footprint of both positive and negative life events on the PFC. In turn, PFC is well known to regulate the future response to stimuli and the processing of emotional valence thanks to its top-down controlling action on limbic structures such as the amygdala^19^. Interestingly, the rapid-acting antidepressant ketamine^20^ is known to rescue depressive-like behaviors induced by the CMS protocol^21,22^, an action that is likely related to its established effects on both dopaminergic and glutamatergic transmission^23–25^.

Based on these premises, we decided to investigate the role of DA-dependent synaptic plasticity at medial PFC (mPFC) circuits in the expression of depressive-like behavior induced by CMS: our hypothesis was that the outcome of either DA-LTP or DA-LTD induced in the mPFC of CMS-treated animals with high levels of anhedonia (vulnerable rats) is different compared to both control and resilient CMS-treated rats. In parallel, based on the established role of corticolimbic regions in supporting the stress response and motivation-related behaviors, we investigated whether CMS affects the activity of two major synaptic pathways connecting the mPFC to the basolateral amygdala (BLA): the prevision was that the homeostatic balance of these synaptic is perturbed by CMS, causing synaptic activity to be either increased or reduced, thus compromising the top-down control of PFC onto amygdala. Finally, we hypothesized that acute sub-anesthetic ketamine treatment (10 mg/kg) exerts a rescuing action on the changes produced by chronic stress on the investigated synaptic phenomena.

## Materials and methods

### Animals

Experiments were performed on 85 Sprague–Dawley rats (175–200 g upon arrival; Charles River, Italy; 6 weeks of age). When not subjected to stress protocols (see below), rats (2 per cage) were maintained under a 12 h/12 h light/dark cycle, with food and water ad libitum and constant 23 °C temperature. Rats were habituated to the animal house condition for 4 days before any procedure. All experiments were performed during the light phase. All methods were performed in accordance with relevant guidelines and regulations. Procedures were approved by the Animal Care and Use Committee of San Raffaele Scientific Institute, in accordance with the Italian Ministry of Health (IACUC 905). The study is reported in accordance with ARRIVE guidelines. Subjects were randomly assigned to the experimental group and were excluded from analysis only when experimental data could not be acquired due to technical reasons. Ketamine (10 mg/kg, Ketavet 100, Intervet Production, Aprilia, Italy) was intraperitoneally (i.p.) administered.

### Behavioral procedures

#### Experimental design

Experimental design and allocation of rats to the experimental groups is depicted in the scheme of Supplementary Fig. 1a. After the 4 days of habituation to housing conditions, rats were tested for SPT (see below), then randomly assigned to the control (14 rats) and CMS groups (71). After 5 weeks, all rats were let rest for 48 h (no stress) and then tested again for SPT. At the end, rats were randomly assigned to subgroups for the different data acquisition procedures (electrophysiological recordings, ER; immunostaining, IS; SynaptoZip experiments, SZ; Supplementary Fig. 1a). A subgroup of CMS-treated rats for each data acquisition group was treated with ketamine (instead of vehicle) 1.5 h before sacrifice (as a rest period in the home cage).

#### Sucrose preference test (SPT)

SPT procedure was adapted from^26^. Rats were habituated to 1% sucrose for 24 h, then water and food deprived for 12 h and finally presented with two identical bottles containing either tap water or 1% sucrose for the next 12 h (night phase; food provided during test). Sucrose preference was calculated as: 100 · (sucrose intake [g]/total fluid intake [g]). A second SPT was performed 48 h after the end of CMS (for both stressed and control rats). The threshold for separating resilient from chronic rats (90%) was equal to the mean minus one standard deviation of the control group.

#### Unpredictable chronic mild stress (CMS)

The CMS protocol was implemented as previously described^22,27,28^ and includes a set of mild stressors that are applied in a random sequence to minimize habituation, once or twice a day for 5 weeks, with the exclusion of some randomly occurring resting days. Stressors lasted 4–12 h depending on stressor and included: food or water deprivation (12 h) concluded with a short the presentation of an empty bottle or food pellets scattered in the cage as additional stress; wet cage (12 h); isolation (1 rat per cage) or overcrowding (6 rats per cage) in the house cage (6–8 h); intruder in cage (3 rats per cage) (6–8 h); switching couples of rats (2 rats per cage) (6–8 h); forced swim (one 5-min session per week, followed by drying and warming of the rat, see below; forced swim was never introduced after food or water deprivation); cage tilting to 45° (12 h); light/dark cycle disruption (24 h of dark or 24 h of light or switching light/dark phases). The forced swim session was performed once a week: rats were put for 5 min in a transparent plexiglass cylinder (60 cm high, 25 cm diameter) filled with room temperature water, then rapidly warmed/dried. The entire session was videotaped and analyzed using EthoVision XT14 software (https://www.noldus.com/ethovision) by an experimenter blind to the experimental condition. No stressors were delivered to rats during the 48 h before SPT or other experimental procedures.

### Acute slices preparation

Brain slices were obtained from control and CMS rats as previously described^29^. Briefly, rats were deeply anesthetized with sevoflurane and then injected intraperitoneally with a lethal Thiopental dose (50 mg; RotexMedica, GMBH, Germany). The experimenter performing brain slicing and recording was blinded to the experimental group. Rats were then transcardially perfused with ice-cold carbonated ACSF mixed with 5000 IU l^−1^ heparin (Pharmatex, Milano, Italy), containing (in mM) 119 NaCl, 2.5 KCl, 1 NaH_2_PO_4_, 26.2 NaHCO_3_, 1 CaCl_2_, 3 MgCl_2_, 11 d-glucose, while the head was cooled by means of ice application. After decapitation, brains were quickly removed and transferred to an ice-cold cutting solution. Coronal slices containing mPFC were obtained using a Vibratome (2.5–3.5 mm anterior to bregma, slice thickness 400 μm; Vibratome Series 1000, TPI, St Louis, MO, USA).

### Electrophysiological recordings

Slices were transferred to the recording chamber and constantly perfused with ACSF (290 mOsm, room temperature for LTD experiments and 32 °C for LTP experiments) at a rate of 2 ml/min and bubbled with 95% O_2_/5% CO_2_ gas mixture. Extracellular recordings were obtained by a glass electrode (resistance 0.3–0.7 MΩ) filled with ACSF and placed on layer V of the mPFC, where the basal dendrites and cell body of pyramidal neurons are located. Constant current stimuli (100–300 μA) were delivered by tungsten bipolar electrodes positioned on layer II–III of the same area to evoke fEPSPs. The testing stimuli for the basal synaptic response was delivered at 0.01 Hz (50 μs pulse duration). LTP experiments were performed in the presence of 1 μM picrotoxin. To induce LTP, 5 trains of 300 Hz tetanus (0.5 s train duration, 50 μs pulse duration, 3 min inter-train interval) were used, after bath application of DA (Sigma-Aldrich; 25 μM for 10 min). To induce LTD, 3 Hz stimulation for 15 min was applied in the presence of DA (200 μM, applied 10 min before start of stimulation and till 5 min after its end). DA (always mixed with ascorbic acid 5–20 μM to prevent oxidation) was locally applied by the means of a thin cannula at a rate of ~ 1 mL/min located above the layer V. For quantification of both LTP and LTD, final fEPSP amplitude (normalized using the baseline) was averaged 30–40 min after the end of electrical stimulation. Whole-cell patch-clamp recordings were obtained with voltage-clamp configuration (Axopatch 200B amplifier; Axon Instruments, Foster City, CA) on the same slice preparations described above, using ACSF adjusted to 295 mOsm and supplemented with 100 μM picrotoxin. Patch pipette electrodes (resistance 5–10 MΩ) were filled with an intracellular solution containing (in mM): 110 d-gluconic acid, 5 MgCl_2_, 10 NaCl, 0.6 ethylene glycol-bis(2-aminoethylether)-*N*,*N*,*N*,*N*-tetraacetic acid (EGTA), 2 ATP, 0.2 GTP, 49 HEPES, 5 QX-314 and 0.1 spermine (pH adjusted to 7.2 with CsOH; osmolarity 285 mOsm). Membrane and series resistances were constantly monitored by applying 5 mV depolarizing pulses (2recordings with series resistance changing more than 20% were discarded). After obtaining a stable patch, excitatory post-synaptic currents (EPSCs) were evoked using a bipolar electrode positioned in layer II–III (constant current pulses of 50–500 μA). AMPA/NMDA ratio and rectification index were obtained using a procedure inspired by previous reports^30^. Analysis of recordings was performed with custom code developed in MATLAB (Mathworks).

### Viral vectors and peptides

To obtain simultaneous analysis of two different synaptic populations in the same animal, we developed a red-shifted fluorescent variant of the SynaptoZip (SZ) protein^31^, by standard restriction cloning. Briefly, the coding sequence of mCherry was derived from the pmCherry-C1 plasmid (Clonetech, Takara Bio Group SAS, France), then adapted using an 80 bp synthetized gene (Eurofins Genomics Srl, Italy) for in-frame fusion with SZ and multiple cloning site (MCS) replacement. Then, the mCherry-SynaptoZip sequence was inserted in the lentiviral vector at AgeI-SalI sites^32^. The final plasmid was verified by DNA sequencing (Eurofins Genomics Srl, Italy) and viral particles (titers ~ 10^9^ TU ml^−1^) were produced as previously described^32^.

The SB peptide (CGGAQLKKKLQALKKKNAQLKWKLQALKKKLAQ) was produced by synthesis (JPT Peptide Technologies GmbH, Berlin, Germany) and conjugated to an Alexa Fluor 647 (Thermo Fisher Scientific). Plasmids with full sequences are available at Addgene with the following IDs: https://www.addgene.org/122523/ (pLenti-hPGK-eGFP-SynaptoZip) and https://www.addgene.org/177317/ (pLenti-hPGK-mCherry-SynaptoZip).

### Stereotaxic surgery

Rats were subjected to stereotaxic surgery for the delivery of the viruses carrying mCherry-SynaptoZip (RZ) in the prelimbic cortex (PL: AP 3; ML 0.6; DV 3.4; volume 2 μL) and eGFP-SynaptoZip (GZ) in the infralimbic cortex (IL: AP 2.6; ML 0.6; DV 5; volume 2 μL). Anesthesia was maintained by 3.5% sevoflurane using mechanical ventilation and standard surgical care was adopted as previously described^31,33^. Briefly, animals were treated with gentamicin to prevent infections (1.5 mg kg^−1^ i.p.; Gentamycin Sulphate, Italfarmaco). The viral vector was delivered using glass micropipettes (60 μm tip diameter; borosilicate, VWR International) and a micro-perfusion system (100 nl min^−1^; Harvard Apparatus Pump 11 Elite). After delivery, the pipette was left in place for 10 min. At the end of delivery, sutures were placed under anesthesia, the animal returned to cage and the recovery and health state monitored. To perform SB uptake experiments (after the final SPT), Synbond-Alexa647 (SB) was delivered to the BLA of anesthetized rats using the same surgery procedure described above (BLA: AP 2.7; ML 5; DV 8.4; volume 2 μL of SB at 1 μM). After recovery from surgery, rats were kept in their home-cage for 90 min (fixed time window for SB uptake).

### Tissue collection

For collecting brains for tissue analysis, animals were sacrificed with a lethal dose of Thiopental (Thiopental, Vuab-Pharma) and then intracardially perfused with cold saline supplemented with heparin (5000 IU l^−1^), followed by PFA 4% in 120 mM phosphate buffer (pH 7.4 at 4 °C). The brain was then removed, submerged in PFA O/N (4 °C), embedded in 4% agar for cutting 35–40 μm thick slices using a vibratome (VT1000S, Leica, Germany). Blood samples were acquired before sacrifice and corticosterone serum concentration was measured using a commercial kit (Corticosterone EIA kit from Oxford Biomedical).

### Immunostaining

PFA fixed brain sections were quenched (0.1 M glycine, 120 mM phosphate buffer, pH 7.4, 30 min, 4 °C), and permeabilized in blocking buffer (3% Triton X-100; 120 mM phosphate buffer, 1% BSA, pH 7.4, 1 h, 4 °C). Samples were then incubated with primary antibodies (1:200 v/v in blocking buffer; O/N at 4 °C) followed by secondary antibodies (1:200 v/v in blocking buffer, 1–2 h at 23 °C). Samples were washed with blocking buffer (4 °C), rinsed in PBS, and mounted (FluorSave, EMD Millipore). Primary antibodies used were anti-cFOS (rabbit sc-52, SantaCrutz) and anti-NeuN (mouse IgG1 clone A60, EMD Millipore). Secondary antibodies used were: AlexaFluor647 donkey anti-rabbit IgG (H + L) and FITC goat anti-mouse IgG (H + L) (Jackson ImmunoResearch). In some cases, slices were loaded with DAPI (1 μL/mL).

### Image collection and analysis

For analysis of SZ experiments and cFOS expression, fluorescence images were collected from brain sections using confocal microscopes (LSM510, Carl Zeiss, Jena, Germany; Fluoview FV3000, Olympus, Japan). Low magnification images were acquired using a 10 × 0.4 NA air objective. High magnification images for SZ experiments were acquired using either a 100 × 1.4 NA or 60 × 1.4 oil objective. cFOS/NeuN images were acquired using either 40 × 0.8 NA or 30 × 0.8 NA oil objectives. All acquisition parameters were kept fixed among compared experiments. Images from SZ experiments were acquired and analyzed as described in^31^. cFOS analysis was similar to previous reports^34^. All numerical analyses were then performed in MATLAB (Mathworks). The representative fluorescence images were processed using ImageJ for median filtering and changing colormaps. See supplementary information for details about image analysis.

### Statistical analysis

Samples size for synaptic plasticity experiments and SZ experiments were set based on statistical and ethical considerations as described in^29^ and^31^, respectively. Rats were randomly assigned to the group and the experimenter was blind to the experimental group. For data sets with multiple groups, Kruskal–Wallis test was used for evaluating the significance of main effect of group, followed by post-hoc permutation tests for independent samples (10^4^ permutations; two-tailed) implemented as previously described^35^. Bonferroni–Holm (B–H) correction of *p* values was always applied for multiple comparisons. For repeated measure design (rats’ weight gain and activity during forced swim over time), rANOVA was performed and *p* value for the main effect was corrected for lack of homoscedasticity using Greenhouse–Geisser approximation. Then, post-hoc multiple comparisons were performed using the Scheffé S procedure. When required, normal distribution of data was verified on Q–Q plots or with the Kolmogorov–Smirnov (K–S) test. For the statistical analysis of synaptic SB uptake and cFOS expression we linear generalized mixed models (GLMMs) and then either ANOVA or a likelihood test was performed on the GLMM followed by analysis of all contrasts and B–H correction (for multiple groups) of the obtained *p* values. All details about model formulation and power analysis can be found in the supplementary methods of Supplementary information.

All ` representation were performed using MATLAB (Mathworks). All descriptive statistics provided are mean ± SE. Threshold for statistical significance was set to 0.05 and indicated on graphs as follows: *p < 0.05; **p < 0.01; ***p < 0.001; ****p < 0.0001.

Additional details about all procedures can be found in the Supplementary information file.

## Results

### CMS induces anhedonia in a subgroup of stress-vulnerable rats

**Figure 1:**
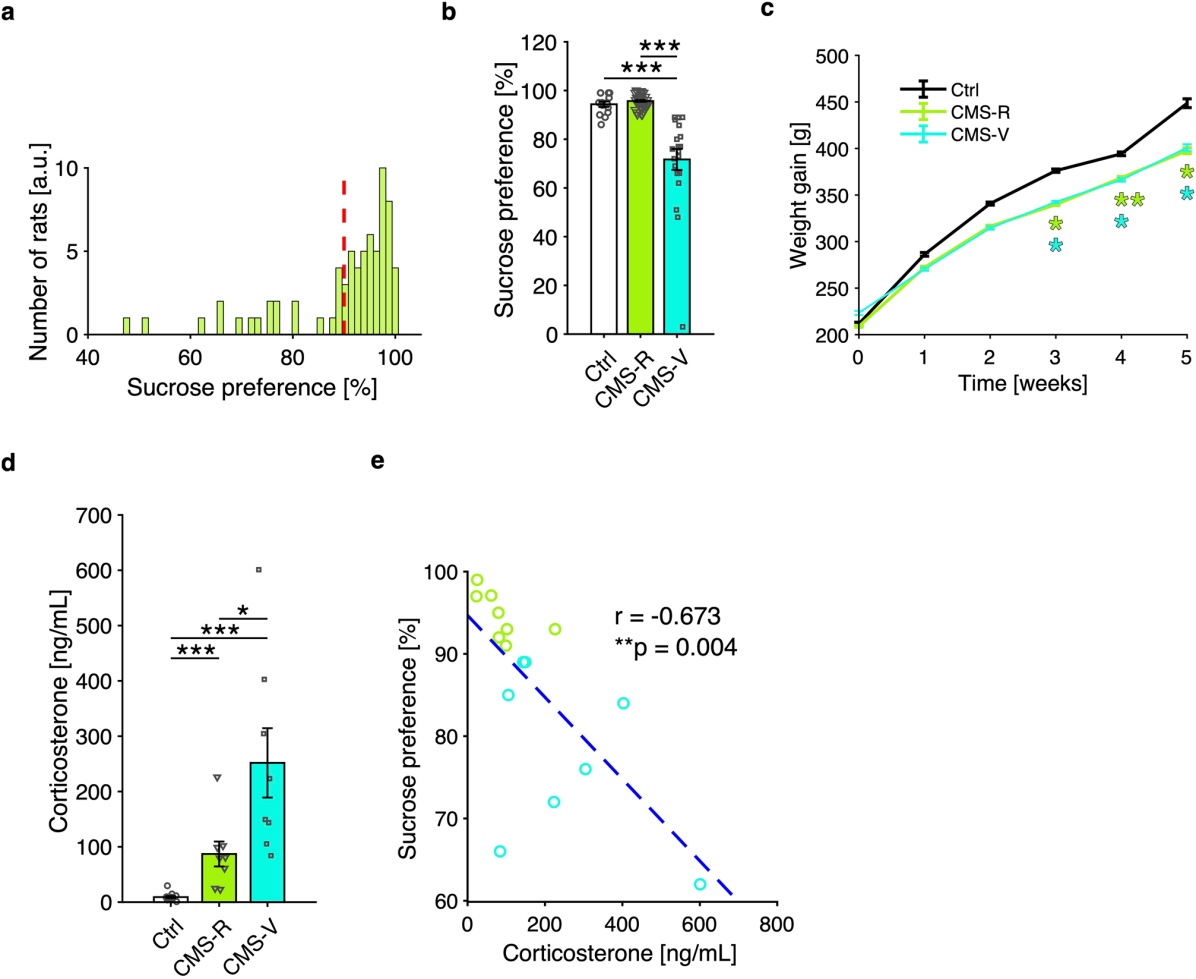
Effects of CMS protocol on sucrose preference, weight gain and serum levels of CORT. (**a**) Distribution of sucrose preference and 90% preference threshold (dashed red line). (**b**) CMS reduces sucrose preference in the SPT in vulnerable (CMS-V) rats if compared to either control (Ctrl) or resilient (CMS-R) rats (n: Ctrl/CMS-R/CMS-V = 14/50/21; K–W test, *p* < 10^−4^; permutation tests, B–H correction: CMS-R vs. Ctrl, *p* = 0.1851; CMS-V vs. Ctrl, *p* = 0.0003; CMS-V vs. CMS-R, *p* = 0.0003). (**c**) CMS reduced weight gain in both CMS-R and CMS-V rats from week 3 on (rANOVA: Time F(5,110) = 372.71, *p* < 10^−4^; Time x Group F(10,110) = 3.162, *p* = 0.040; post-hoc analysis with Scheffé's S procedure: week 3, CMS-R vs. Ctrl, *p* = 0.011, CMS-V vs. Ctrl, *p* = 0.023; week 4, CMS-R vs. Ctrl, *p* = 0.004, CMS-V vs. Ctrl, *p* = 0.023; week 5, CMS-R vs. Ctrl, *p* = 0.016; CMS-V vs. Ctrl, *p* = 0.049; all other comparisons n.s.). (**d**,**e**) CORT levels are higher for CMS-V rats compared to CMS-R rats, as well as for each of the two groups compared to controls (**d**) (n: Ctrl/CMS-R/CMS-V = 14/8/8; K–W test, *p* < 10^−4^; permutation tests, B–H correction: CMS-R vs. Ctrl, *p* = 0.0003; CMS-V vs. Ctrl, *p* = 0.0003; CMS-V vs. CMS-R, *p* = 0.014), with a significant inverse correlation between sucrose preference in the SPT and CORT (**e**) (Pearson’s correlation: r = − 0.673; *p* = 0.004).

After 4 days of habituation to the housing conditions, rats were tested for sucrose preference (SPT) and then randomly assigned to the CMS protocol which lasted 5 weeks; at the end, a second SPT was carried out. In our conditions, SPT resulted in very high levels of sucrose preference in control animals (94.4 ± 4.15%; mean ± SD). The whole dataset of preference distribution is reported in Fig. 1a. For separating resilient (CMS-R) from vulnerable rats (CMS-V), we set a discrimination threshold at 90% (equal to mean—1SD of controls preference): using this parameter, we found that 21 out of the 71 rats exposed to CMS for 5 weeks displayed behavioral vulnerability to CMS (n: Ctrl/CMS-R/CMS-V = 14/50/21; K–W test, *p* < 10^−4^; permutation tests, B–H correction: CMS-R vs. Ctrl, *p* = 0.1851; CMS-V vs. Ctrl, *p* = 0.0003; CMS-V vs. CMS-R, *p* = 0.0003) (Fig. 1b).

CMS also induced a significant and remarkable reduction of weight gain of comparable degree in both CMS-R and CMS-V rats compared to controls, starting from week 3 (rANOVA: Time F(5,110) = 372.71, *p* < 10^−4^; Time × Group F(10,110) = 3.162, *p* = 0.040; post-hoc analysis with Scheffé's S procedure: week 3, CMS-R vs. Ctrl, *p* = 0.011, CMS-V vs. Ctrl, *p* = 0.023; week 4, CMS-R vs. Ctrl, *p* = 0.004, CMS-V vs. Ctrl, *p* = 0.023; week 5, CMS-R vs. Ctrl, *p* = 0.016; CMS-V vs. Ctrl, *p* = 0.049; all other comparisons n.s.) (Fig. 1c).

Since one forced swim session was weekly included in the CMS as a stress episode, we analyzed rats’ swimming behavior: no significant difference was found between CMS-R and CMS-V rats, but both groups showed a significant increase of inactivity (rANOVA: Time F(4,44) = 3.826, *p* = 0.0253; Time × Group, F(4,44) = 0.785, n.s.; Supplementary Fig. 1b) and a parallel decrease in moderate (rANOVA: Time F(4,44) = 5.162, *p* = 0.007; Time × Group, F(4,44) = 0.781, n.s.; Supplementary Fig. 1c) and high (rANOVA: Time F(4,44) = 3.449, *p* = 0.048; Time × Group, F(4,44) = 1.958, n.s.; Supplementary Fig. 1d) levels of activity over weeks, which is suggestive of increased levels of behavioral despair^36^.

In addition, we found that the serum levels of the stress hormone corticosterone (CORT) were significantly different among all groups (n: Ctrl/CMS-R/CMS-V = 14/8/8; K–W test, *p* < 10^−4^; permutation tests, B–H correction: CMS-R vs. Ctrl, *p* = 0.0003; CMS-V vs. Ctrl, *p* = 0.0003; CMS-V vs. CMS-R, *p* = 0.014) (Fig. 1d), with CORT level of CMS rats being inversely correlated to sucrose preference (Pearson’s correlation: r = − 0.673; *p* = 0.004) (Fig. 1e), thus supporting a significant direct relationship between experienced stress and the expression of anhedonia levels in CMS-treated rats.

### DA-LTP, but not DA-LTD, is occluded in rats vulnerable to CMS

We previously reported that acute mild stress affects a form of LTP that can be induced in mPFC^29^ and is strongly dependent on DA action (DA-LTP). Both synaptic potentiation (DA-LTP) and depression (DA-LTD) have been widely characterized^12,37^ in vitro, using several recording and stimulation approaches, including high-frequency stimulation (HFS) and spike-timing-dependent plasticity (STDP). The common denominator is the need for DA to be provided to tissue at the induction phase, with specific timing: albeit the effect of DA administration alone is transient, when paired with electrical stimulation the result is a stable potentiation or depression of synaptic terminals, depending on DA concentration and stimulus frequency.

**Figure 2:**
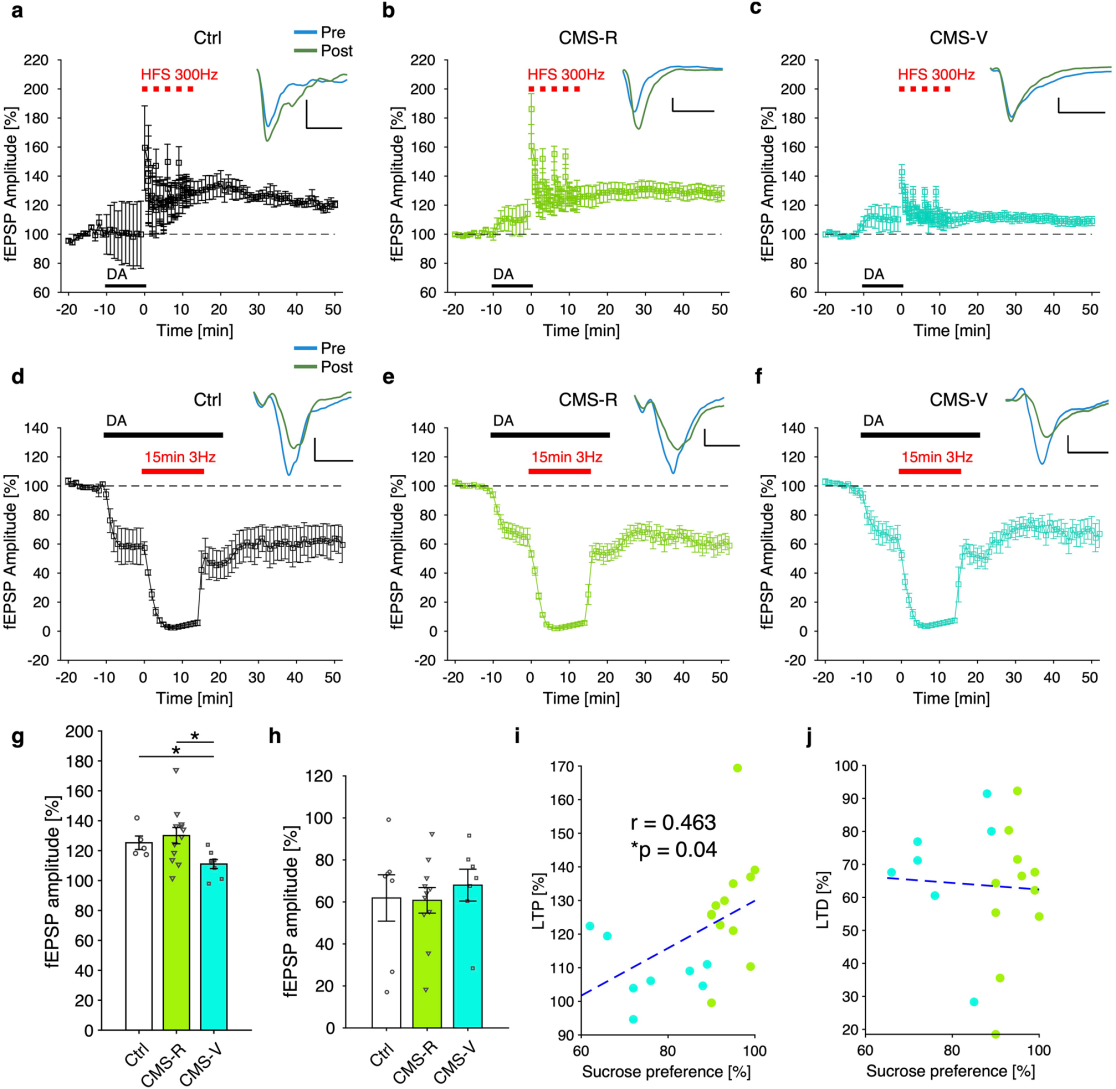
DA-LTP is occluded in CMS-vulnerable rats while DA-LTD is unaffected. (**a**–**c**) Time course of DA-LTP experiments in mPFC slices coming from control (**a**), CMS-R (**b**) and CMS-V rats (**c**); representative fEPSP responses before (blue) and after (green) LTP induction are shown on top of each graph (scale bar: 20 ms × 0.2 mV). (**d**,**e**) Time course of DA-LTD experiments in mPFC slices coming from control (**d**), CMS-R (**e**) and CMS-V rats (**f**); representative fEPSP responses before (blue) and after (green) LTD are shown on top (scale bar: 20 ms × 0.2 mV). (**g**,**h)** Analysis of population data shows significant effect of group for DA-LTP induction (n: Ctrl/CMS-R/CMS-V = 5/12/8 slices from 5/12/8 rats; K–W test, *p* = 0.019; permutation tests, B–H correction: CMS-R vs. Ctrl, n.s.; CMS-V vs. Ctrl, *p* = 0.036; CMS-V vs. CMS-R, *p* = 0.034) (**g**), while lack of effect for DA-LTD experiments (n: Ctrl/CMS-R/CMS-V = 7/11/7 slices from 7/11/7 rats; K–W test, n.s.; permutation tests, B–H correction: no comparison resulted significant) (**h**). (**I**,**j**) Final fEPSPs amplitude resulted correlated to sucrose preference after DA-LTP (Pearson’s correlation: r = 0.463; *p* = 0.04) (**i**), but not after DA-LTD (Pearson’s correlation: r = − 0.051; n.s.; dashed blue lines represents 2 parameters linear fitting) (**j**). *DA* dopamine, *HFS* high-frequency stimulation.

Since CMS protocol is based on the administration of mild stressors in a random sequence and produces a downregulation of the DA system^6,38^ likely underlying the expression of depressive-like behavior, we hypothesized that DA-LTP might be altered in rats vulnerable to CMS. To test this hypothesis, we collected acute brain slices including the mPFC from control and CMS rats immediately after the SPT. As shown in Fig. 2a,b, DA-LTP was reliably induced in slices coming from both control (Fig. 2a) and CMS-R (Fig. 2b) rats and lasted for at least 50 min after the start of high-frequency stimulation (HFS). On the contrary, DA-LTP was almost completely occluded in CMS-V rats (Fig. 2c), and significantly reduced compared to both control and CMS-R rats (n: Ctrl/CMS-R/CMS-V = 5/12/8 slices from 5/12/8 rats; K–W test, *p* = 0.019; permutation tests, B–H correction: CMS-R vs. Ctrl, n.s.; CMS-V vs. Ctrl, *p* = 0.036; CMS-V vs. CMS-R, *p* = 0.034) (Fig. 2g).

We also tested DA-LTD^29^ in slices from the same groups: strong LTD (~ 40% reduction) was reliably obtained in control (Fig. 2d), CMS-R (Fig. 2e) and CMS-V (Fig. 2f) rats, and no significant group effect was detected (n: Ctrl/CMS-R/CMS-V = 7/11/7 slices from 7/11/7 rats; K–W test, n.s.; permutation tests, B–H correction: no comparison resulted significant) (Fig. 2h). Finally, while potentiation level obtained in LTP experiments resulted correlated to sucrose preference (Pearson’s correlation: r = 0.463; *p* = 0.04) (Fig. 2i), depression level after DA-LTP was not (Pearson’s correlation: r = − 0.051; n.s.) (Fig. 2j).

Hence, in our experiments, CMS protocol caused a strong and significant occlusion of DA-dependent LTP selectively in CMS-V rats, while DA-LTD is unaffected by stress. In addition, the degree of LTP occlusion seems directly correlated to the level of rat’s anhedonia.

### Sub-anesthetic ketamine rescues differences in DA-LTP between vulnerable and resilient rats

**Figure 3:**
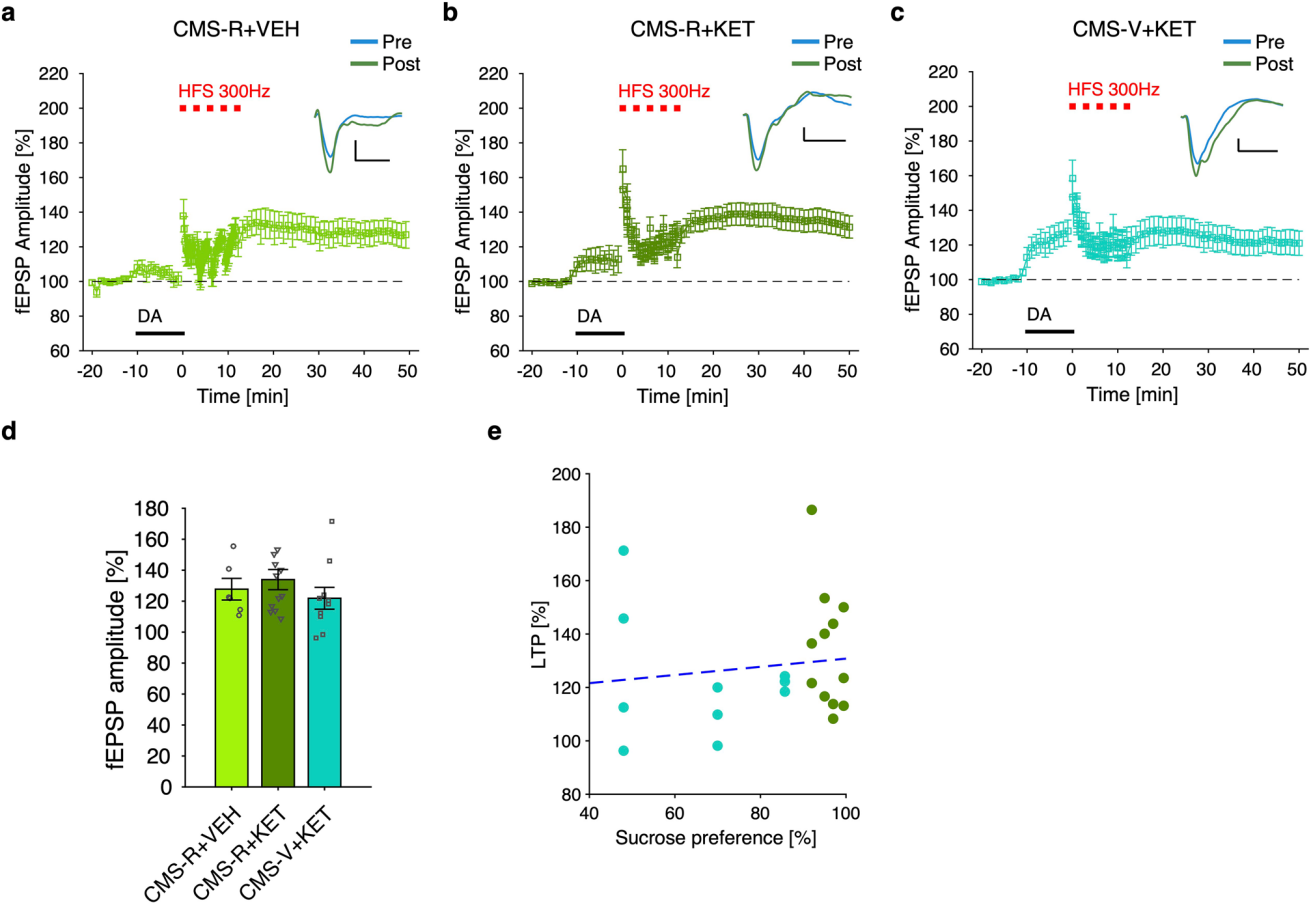
Acute sub-anesthetic ketamine treatment rescues differences in DA-LTP between resilient and vulnerable rats. (**a–c**) Time course of DA-LTP experiments in mPFC slices coming from CMS-R+VEH (**a**), CMS-R+KET (**b**) and CMS-V+KET (**c**) rats; ketamine (KET; 10 mg/kg) was administered 1.5 h before slices production; representative fEPSP responses before (blue) and after (green) DA-LTP are shown on top (scale bar: 20 ms × 0.2 mV). (**d**) Analysis of population data shows no significant difference among the three groups (n: CMS-R+VEH/CMS-R+KET/CMS-V+KET = 6/12/11 slices from 5/4/4 rats; permutation test, B–H correction: n.s.). (**e**) fEPSPs amplitude after DA-LTP was not correlated with sucrose preference in CMS-V+KET rats (Pearson’s correlation: r = 0.230; n.s.). *DA* dopamine, *HFS* high-frequency stimulation.

Based on these results, we decided to test whether the rapid-acting antidepressant ketamine, which is known to activate glutamatergic AMPA receptors at sub-anesthetic dose^25^ and to rescue depressive-like behavior induced by the CMS protocol^21,22^, is also able to rescue the changes of DA-LTP observed in the mPFC of CMS-V rats. To test this hypothesis, a new cohort of CMS rats was SPT-tested and then acutely injected with ketamine (10 mg/kg, i.p.) 1.5 h before the production of brain slices. To reduce the number of animals involved in the study, a subgroup of CMS-R rats was injected with vehicle and used as reference group, while all the CMS-V animals were treated with ketamine. From these animals, a stable DA-LTP was obtained as above (Fig. 3a).

Importantly, administration of ketamine to CMS-R rats did not produce any change in DA-LTP (Fig. 3b). At the same time, CMS-V rats treated with ketamine produced levels of DA-LTP not significantly different from either treated or non-treated CMS-R animals (Fig. 3c), indicating that DA-LTP occlusion by stress is not observed in CMS-V rats treated with acute ketamine (n: CMS-R+VEH/CMS-R+KET/CMS-V+KET = 6/12/11 slices from 5/4/4 rats; permutation test, B–H correction: n.s.) (Fig. 3d). Accordingly, no correlation between LTP level and sucrose preference was detected for the group of CMS+KET rats, indicating that LTP after ketamine is not related to the level of CMS-induced anhedonia (Pearson’s correlation: r = 0.230; n.s.; dashed blue line represents 2 parameters linear fitting) (Fig. 3e). These results suggest that a single sub-anesthetic dose of ketamine acutely rescues differences in DA-LTP between vulnerable and resilient rats.

### Vulnerable rats display higher AMPA/NMDA ratio at mPFC layer V principal neurons

**Figure 4:**
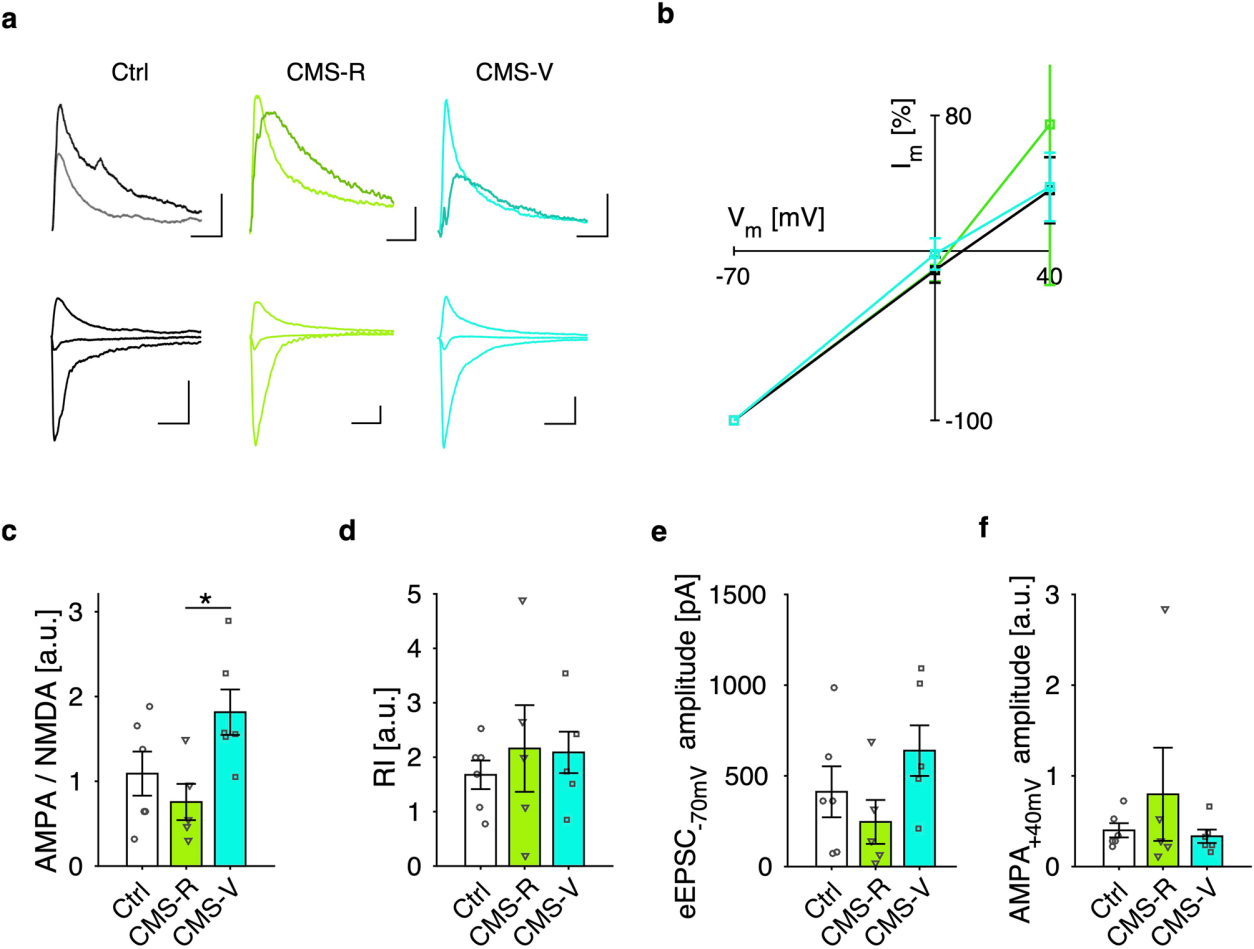
Vulnerability to CMS reflects higher potentiation level at layer V principal neurons. (**a**) Exemplary recordings of evoked EPSCs at layer V PNs for control (black), CMS-R (green) and CMS-V (cyan) rats: NMDA (darker color) and AMPA (lighter color) components a +40 mV holding potential (top traces) (scale bars: 100 ms × 25 pA); AMPA component at −70 mV, 0 mV and +40 mV (bottom traces) (scale bars: 100 ms × 50 pA). (**b**) AMPA I–V relationship (normalized). (**c**) AMPA/NMDA ratio is higher for CMS-V rats compared to CMS-R rats (n: Ctrl/CMS-R/CMS-V = 6/5/6 cells from 3/3/4 rats; K–W test, *p* = 0.036; permutation tests, B–H correction: CMS-V vs. CMS-R, *p* = 0.036), reflecting a higher potentiation level of synapses onto layer V PNs. (**d**–**f)** Rectification index (**d**), absolute EPSC amplitude (at − 70 mV holding potential) (**e**) and normalized AMPA component at +40 mV (**f**) do not vary across groups (same recordings as in h; K–W test, n.s.; permutation tests, B–H correction: n.s.).

Since the level of synaptic strength can alter the metaplastic potential of a synaptic pathway and occlude LTP, we then evaluated synaptic transmission at layer V principal neurons using whole-cell recordings on mPFC slices coming from both control and CMS rats. In a subset of animals, we separated rats based on the absolute change in sucrose preference after CMS using the SPT baseline as reference (CMS-R: increase or no change, CMS-V: reduction; n: Ctrl/CMS-R/CMS-V = 4/7/4; K–W test, *p* = 0.010; permutation tests, B–H correction: CMS-V vs. CMS-R, *p* = 0.013). Cell input resistance and time constant were not found to vary among the three groups (n: Ctrl/CMS-R/CMS-V = 8/7/7 cells from 4/5/4 rats; K–W test, n.s.; permutation tests, B–H correction: n.s.). Currents were then evoked by layer II–III stimulation at different holding potentials, with or without bath application of the NMDAR antagonist APV, to obtain AMPA/NMDA ratio and AMPA I–V curve (Fig. 4a,b). The former was found significantly higher in CMS-V rats compared to CMS-R rats (n: Ctrl/CMS-R/CMS-V = 6/5/6 cells from 3/3/4 rats; K–W test, *p* = 0.036; permutation tests, B–H correction: CMS-V vs. CMS-R, *p* = 0.036) (Fig. 4c), reflecting increased potentiation level of synapses onto layer V PNs. Nevertheless, rectification index (RI) seems constant among the three groups (same recordings of Fig. 4c; K–W test, n.s.; permutation tests, B–H correction: n.s.) (Fig. 4d), suggesting that the observed increase in AMPA/NMDA ratio is not due to the recent insertion of calcium permeable AMPA channels.

Finally, total EPSC amplitude (at − 70 mV holding potential) shows a trend toward increase for CMS-R rats, albeit not significant (same recordings of Fig. 4c,d; K–W test, n.s.; permutation tests, B–H correction: n.s.) (Fig. 4e), while the normalized AMPA component does not vary (same recordings of Fig. 4c–e; K–W test, n.s.; permutation tests, B–H correction: n.s.) (Fig. 4f), consistent with the lack of group effects on RI. In conclusion, an increased AMPA/NMDA ratio is observed in CMS-V rats, suggesting a potential route for the expression of vulnerability to stress, in accordance with the observed occlusion of LTP.

### Neuronal activity in VTA and SN assessed by cFOS expression is promoted by ketamine

DA system is known to be involved in motivation-related behaviors, due to its role in the encoding of incentive salience and in the extrapyramidal control of motor activity. In addition, previous reports have shown that chronic stress downregulates neuronal activity in key DA-producing brain regions such as the VTA^6,38^, contrary to the action of acute forms of stress^29,39–41^.

**Figure 5:**
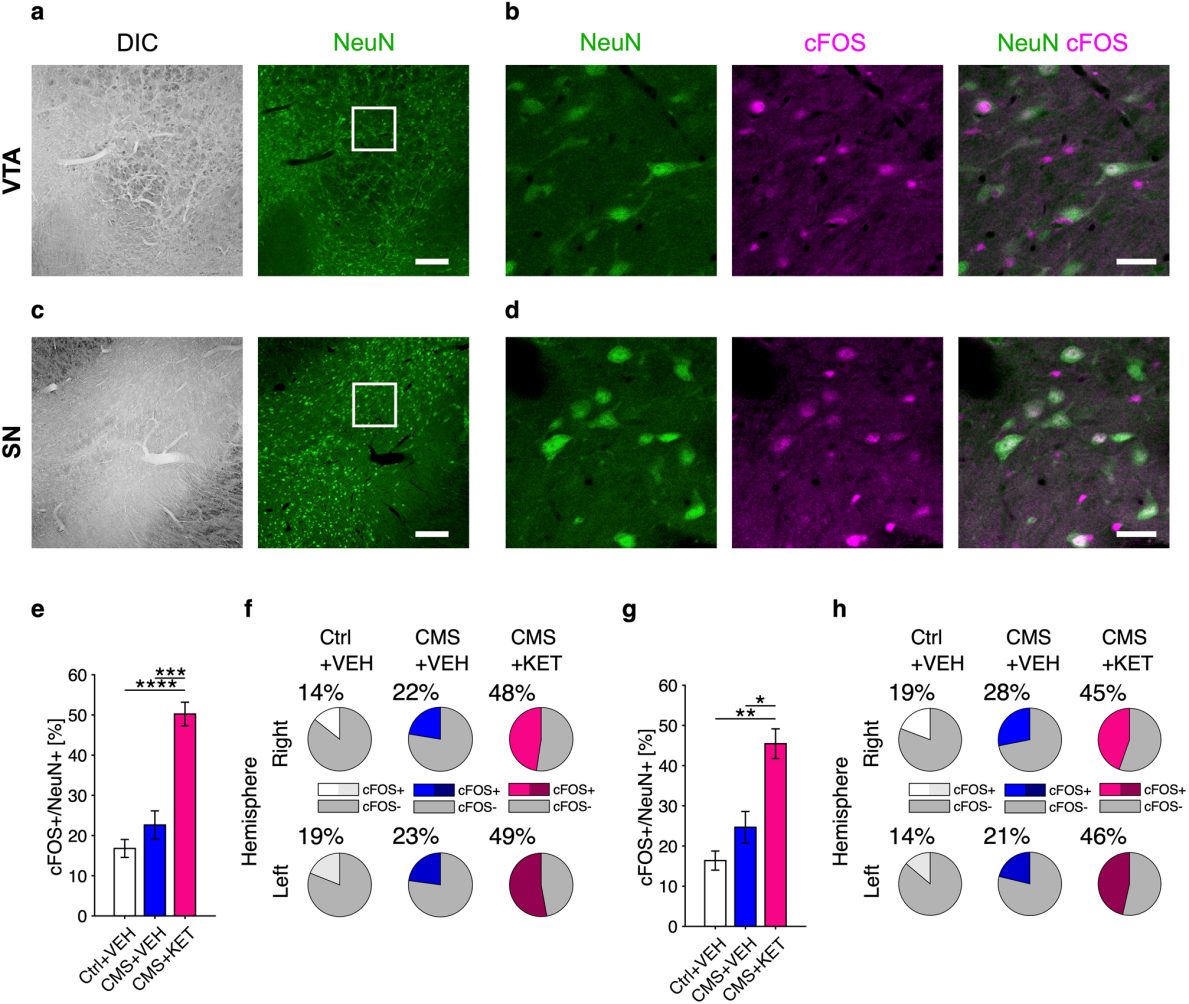
Effects of CMS and ketamine treatment on neuronal activity in the VTA and SN. (**a**–**d**) Exemplary low (**a**,**c**) and high (**b**,**d**) magnification images of VTA (**a**,**b**) and SN (**c**,**d**) stained for NeuN (green) and cFOS (magenta), together with DIC channel (gray) and merge; scale bars: 200 µm (**a**,**c**), 30 µm (**b**,**d**). (**e**) CMS did not significantly affect the relative number of cFOS+/NeuN+ cells in the VTA compared to controls, while ketamine (KET) treatment (10 mg/kg) after CMS produced strong enhancement of VTA activity (n = 88 field-of-views, FOVs, from 2 to 3 rats per group; likelihood ratio test on GLMM model, *p* = 0.002; all contrasts with B–H correction: ﻿CMS+VEH vs. Ctrl+VEH, *p* = 0.229; CMS+KET vs. Ctrl+VEH, *p* < 10^−4^; CMS+KET vs. ﻿CMS+VEH, *p* = 0.0002). (**f**) The relative number (grand average) of cFOS+/NeuN+ cells in the VTA appears as homogeneous between the two hemispheres. (**g**) Similarly to the VTA case, CMS did not significantly increase the relative number of cFOS+/NeuN+ cells in the SN compared to controls, while KET treatment (10 mg/kg) after CMS significantly promotes cFOS expression in the SN (n = 82 FOVs from 2 to 4 rats per group; likelihood ratio test on GLMM model, *p* = 0.0096; all contrasts with B–H correction: CMS+VEH vs. Ctrl+VEH, *p* = 0.432; CMS+KET vs. Ctrl+VEH, *p* = 0.002; CMS+KET vs. CMS+VEH, *p* = 0.012). (**h**) The relative number (grand average) of cFOS+/NeuN+ cells in the SN appears as homogeneous between the two hemispheres.

We then assessed the effect of CMS on the activity of VTA and SN dopaminergic nuclei, with or without the ketamine treatment, to test whether stress and/or ketamine were able to alter dopaminergic activity. To this aim, we performed immunostaining of PFA-fixed brain slices including the VTA (Fig. 5a,b) or the SN (Fig. 5c,d) coming from control treated with vehicle and CMS rats (either vehicle or ketamine treated) to measure the expression of the immediate early gene (IEG) cFOS, an indirect measure of action potential activation^34,42^, in NeuN positive cells (neurons).

As shown in Fig. 5e, the relative number of cFOS+/NeuN+ neurons in the VTA was not affected by the CMS protocol per se; on the contrary, ketamine treatment (10 mg/kg) after CMS resulted in a significant enhancement of VTA activity (n = 88 field-of-views, FOVs, from 2 to 3 rats per group; likelihood ratio test on GLMM model, *p* = 0.002; all contrasts with B–H correction: CMS+VEH vs. Ctrl+VEH, *p* = 0.229; CMS+KET vs. Ctrl+VEH, *p* < 10^−4^; CMS+KET vs. CMS+VEH, *p* = 0.0002) (Fig. 5e). Such result suggests that VTA responds massively to ketamine treatment in CMS rats, indicating a possible dopaminergic route for ketamine antidepressant action. As shown in Fig. 5f, no hemispheric differences could be detected, thus asymmetrical response to ketamine treatment can be excluded.

Consistent with VTA, SN samples did not show any significant effect of CMS alone on the fraction of cFOS+/NeuN+ cells; also in these samples, ketamine (10 mg/kg) after CMS significantly promoted cFOS expression, suggesting that ketamine likely exerts a potentiating action also on SN (n = 82 FOVs from 2 to 4 rats per group; likelihood ratio test on GLMM model, *p* = 0.0096; all contrasts with B–H correction: CMS+VEH vs. Ctrl+VEH, *p* = 0.432; CMS+KET vs. Ctrl+VEH, *p* = 0.002; CMS+KET vs. CMS+VEH, *p* = 0.012) (Fig. 5g). Also for SN samples no differences between the two hemispheres were detected with such analysis (Fig. 5h).

### Synaptic activity balance at corticolimbic circuits is perturbed by CMS and ketamine treatment counteracts this effect

Stress is known to affect several synaptic pathways of the corticolimbic system. In this context, PFC exerts top-down control on basolateral amygdala (BLA)^43^, an action which is central to emotional regulation^44^. On these bases, we wondered if CMS, aside from causing those synaptic changes at mPFC local circuits described above, were also able to perturb the physiological balance at synaptic circuits connecting the mPFC to the amygdala, potentially contributing to the maintenance of the behavioral phenotype.

To test this hypothesis, we used the synaptic activity sensor SynaptoZip^31^ to measure synaptic activity in vivo at pathways connecting two major divisions of the mPFC, namely the infralimbic (IL) and prelimbic (PL) cortexes, to the BLA. SynaptoZip is a genetically encoded probe that was developed by fusing one component of a highly stable dimeric system (Zip) to the intraluminal end of VAMP-2: upon each round of exo-endocytosis occurring in vivo, SynaptoZip binds and accumulates a small fluorescently labelled peptide, Synbond, which is delivered to brain tissue: synapses are then stably stained proportionally to their exo-endocytosis activity and the signal can be acquired and quantified on fixed tissue.

**Figure 6:**
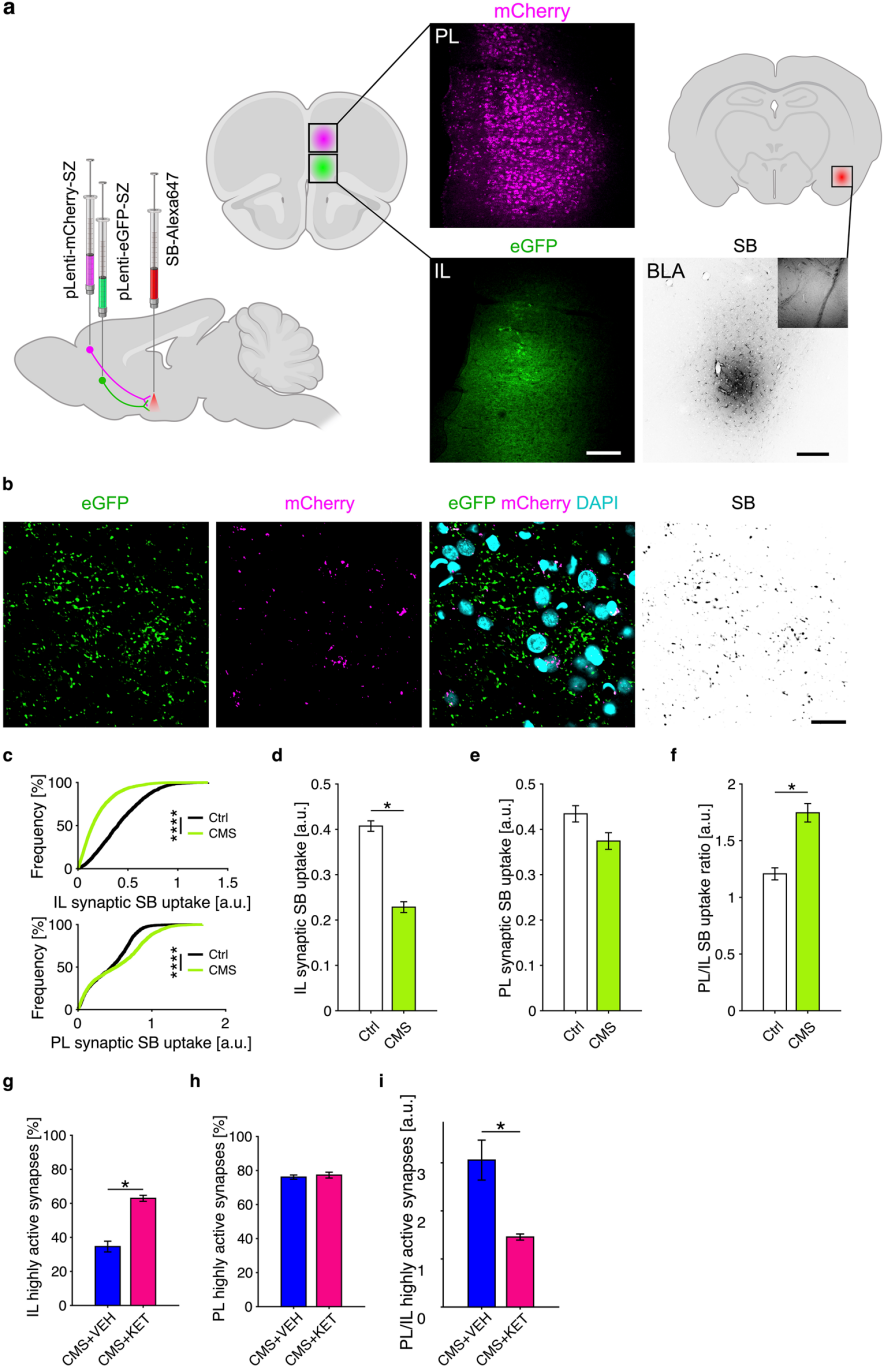
Synaptic activity balance at corticolimbic circuits is perturbed by CMS and ketamine counteracts this effect. (**a**) Left: scheme of the experimental set-up: viruses carrying eGFP-SynaptoZip (green) and mCherry-SynaptoZip (magenta) were delivered to the infralimbic (IL) and prelimbic (PL) mPFC; Synbond-Alexa647 (SB) was delivered to the basolateral amygdala (BLA) at the time of experiment. Right: expression of SZ variants in mPFC (top: PL; bottom: IL) and SB diffusion in the BLA (bottom right; inverted gray colormap; the inset shows DIC channel); scale bars: 200 µm. (**b**) High magnification images of an exemplary BLA field-of-view (FOV) showing eGFP-SynaptoZip (green) and mCherry-SynaptoZip (magenta) expression at synaptic boutons (merge: DAPI channel in cyan) and the related SB uptake (inverted gray colormap); scale bar: 20 µm. **c)** Distribution of synaptic SB uptake from pooled data (top, IL, permutation test, CMS vs. Ctrl, *p* < 10^−4^; bottom, PL, permutation test, *p* < 10^−4^). (**d**) Reduction of IL SB uptake in CMS rats was found using FOV-averaged analysis (IL, n: Ctrl/CMS = 137/195 FOVs from 4/8 rats; ANOVA on GLMM: F(1,330) = 5.028, *p* = 0.026). (**e**) SB uptake at PL-to-BLA boutons seems reduced for CMS rats, but not significantly (PL, n: Ctrl/CMS = 115/144 FOVs from 4/8 rats; ANOVA on GLMM: F(1,257) = 2.659, n.s.). (**f**) Ratio of SB uptake between PL and IL boutons (same FOV) is significantly increased in CMS rats (PL/IL, n: Ctrl/CMS = 115/144 FOVs from 4/8 rats; ANOVA on GLMM: F(1,257) = 9.962, *p* = 0.0018). (**g–i**) The fraction of highly active synapses from IL was significantly increased by ketamine (KET; 10 mg/kg) treatment (**g**), IL, n: CMS+VEH/CMS+KET = 94/201 FOVs from 6/7 rats; ANOVA on GLMM: F(1,293) = 6.156, *p* = 0.014), while it was left unchanged for PL-to-BLA synapses (**h**), PL, n: CMS+VEH/CMS+KET = 119/136 FOVs from 6/6 rats; ANOVA on GLMM: F(1,253) = 1.451, n.s.). Accordingly, the ratio index was found significantly reduced by KET (**i**), PL/IL, n: CMS+VEH/CMS+KET = 78/112 FOVs from 6/5 rats; ANOVA on GLMM: F(1,188) = 5.183, *p* = 0.024).

Figure 6a depicts the experimental set-up. To simultaneously measure activity at both PL-to-BLA and IL-to-BLA pathways, we injected two lentiviruses carrying different fluorescent versions of SynaptoZip (SZ) in either the IL (eGFP-SynaptoZip, green in Fig. 6a scheme) or the PL (mCherry-SynaptoZip, magenta in Fig. 6a scheme; see “Materials and methods” for details on the production of this red-shifted SZ version). This procedure was carried out after the first SPT and before starting the CMS protocol, on both control and CMS rats, so that good and stable SZ expression could be obtained during the protocol timeframe. Then, after performing the final SPT, rats from both groups were injected in the BLA with the Synbond peptide to label spontaneously active mPFC-to-BLA boutons (SB-Alexa647, red in Fig. 6a scheme, inverted gray colormap in microscopy images), and left freely moving in their home cage for 1.5 h.

Rats were then sacrificed and images were collected from PFA-fixed brain sections. Supplementary Fig. 2 shows magnifications of BLA sections where a similar pattern of expression can be appreciated for both eGFP-SynaptoZip (green, Supplementary Fig. 2a) and mCherry-SynaptoZip (magenta, Supplementary Fig. 2b) at presynaptic boutons on axons arriving in the BLA, together with the high level of SB colocalization (cyan). Figure 6b shows a typical FOV obtained in the BLA, where boutons of intermingled axons coming from either IL (eGFP) or PL (mCherry) can be easily distinguished. SB fluorescence (inverted gray, Fig. 6b) was quantified in segmented boutons to obtain synaptic SB uptake (see “Materials and methods” for details). Supplementary Figure 3 shows exemplary 2D (Supplementary Fig. 3a) and 3D (Supplementary Fig. 3b) images obtained from confocal stacks captured at BLA sections, where presynaptic boutons aligned along putative axons can be identified for both populations, with matched pattern of SB labelling.

By looking at pooled data of synaptic SB uptake coming from all synapses analyzed in each group, an effect of CMS treatment on both PL- and IL- to-BLA synapses could be appreciated, indicating that chronic stress is likely affecting PFC synaptic drive onto the BLA (Fig. 6c**,** top graph, IL, n: Ctrl/CMS = 1904/3975; permutation test, CMS vs. Ctrl, *p* < 10^−4^; bottom graph, PL, n: Ctrl/CMS = 1297/2718; permutation test, *p* < 10^−4^). To account for dependance among different field-of-views (FOVs) and rats, data were then FOV-averaged and GLMM models were fitted (see materials and methods for details). With this analysis, significant reduction of IL-to-BLA SB uptake in CMS rats was found (Fig. 6d; IL, n: Ctrl/CMS = 137/195 FOVs from 4/8 rats; ANOVA on GLMM: F(1,330) = 5.028, *p* = 0.026). On the contrary, although a slight reduction of SB uptake at PL-to-BLA boutons was observed for CMS rats compared to controls, this effect was not found significant (Fig. 6e; PL, n: Ctrl/CMS = 115/144 FOVs from 4/8 rats; ANOVA on GLMM: F(1,257) = 2.659, n.s.).

Accordingly, when the ratio of SB uptake between PL and IL boutons in the same FOV (and thus in the same rat) was analyzed, a significant increase was detected for CMS rats (Fig. 6f; PL/IL, n: Ctrl/CMS = 115/144 FOVs from 4/8 rats; ANOVA on GLMM: F(1,257) = 9.962, *p* = 0.0018). Due to the higher power of internal comparisons between the two pathways, such analysis clearly indicates that chronic stress perturbs the balance of synaptic activity at corticolimbic projections toward the amygdala. Since in our experimental conditions (freely moving animal with 1.5 h experimental epoch) almost all synapses were found to be active (SB uptake > 0), we implemented a threshold analysis to quantify the fraction of highly active synapses (threshold: median(SB)/2; see “Materials and methods” for details) from each population (PL and IL), as well as the ratio between the two fractions. This analysis produced comparable results when applied to the CMS and Ctrl data reported above, with the same effects directions (data not shown; IL: ANOVA on GLMM: F(1,340) = 7.542, *p* = 0.006; PL: ANOVA on GLMM: F(1,276) = 1.218, n.s.; PL/IL, ANOVA on GLMM: F(1,243) = 4.69, *p* = 0.031).

We then tested, using the same threshold analysis, if the observed difference in the recruitment of the IL and PL pathways by CMS might be affected by sub-anesthetic ketamine (10 mg/kg) treatment, administered right before starting the 1.5 h SB labelling session. We found that ketamine treatment, compared to vehicle only, significantly increased the fraction of highly active IL synapses in CMS rats (Fig. 6g; IL, n: ﻿CMS+VEH/CMS+KET = 94/201 FOVs from 6/7 rats; ANOVA on GLMM: F(1,293) = 6.156, *p* = 0.014) while it left the fraction of highly active PL synapses unchanged (Fig. 6h; PL, n: ﻿CMS+VEH/CMS+KET = 119/136 FOVs from 6/6 rats; ANOVA on GLMM: F(1,253) = 1.451, n.s.). Accordingly, when we computed the ratio of the PL and IL fractions, respectively, this appeared significantly reduced by ketamine treatment (Fig. 6i; PL/IL, n: CMS+VEH/CMS+KET = 78/112 FOVs from 6/5 rats; ANOVA on GLMM: F(1,188) = 5.183, *p* = 0.024). Hence, these results suggest that acute sub-anesthetic ketamine treatment (10 mg/kg) might counteract the changes produced by CMS on synaptic activity at PFC-to-BLA circuits.

## Discussion

In this work, we investigated the relationship between the induction of a depressive-like behavior, namely anhedonia, by chronic stress and the expression of DA-dependent forms of synaptic plasticity in the mPFC. We found that DA-LTP is occluded in rats vulnerable to CMS, and that this likely reflects differential expression of AMPA receptors. We also showed that such occlusion in plasticity is not observed when rats are treated with acute sub-anesthetic ketamine, known to produce antidepressant effects in both human patients and animals^20–22^. In addition, we investigated cortico-limbic synaptic transmission in vivo, finding that chronic stress alters the activity of pathways connecting the mPFC to the BLA, and that ketamine counteracts these synaptic alterations. Our data also suggest that ketamine rescuing actions on both mPFC plasticity and cortico-limbic synaptic drive are likely mediated by strong promotion of VTA activity.

Several reports in the literature support the idea that both level and timing of stress determine its physiological effects on synaptic plasticity. Deleterious effects of mild and acute stressors on LTP have been mainly observed in cortico-limbic regions^45–48^, but also facilitation has been found at least in the short-term^49^, likely due to the non-genomic action of corticosteroids^50^. In a previous report we showed that also the DA-dependent LTP in the mPFC is enhanced by acute stress^29^. On the other hand, it is accepted that chronic forms of stress, as well as heavy stressors, almost exclusively produce deleterious effects on synaptic plasticity. This is especially true for PFC connectivity and structural plasticity (see^51^ for review), but also for functional plasticity in limbic structures such as the hippocampus and the amygdala, namely suppression of LTP and promotion of LTD (for a review, see^52^).

Unfortunately, studies addressing the effects of chronic stress on mPFC functional plasticity (i.e. LTP/LTD) are still very limited, since most reports focused on a specific afference (e.g. from thalamus^53^ and hippocampus^54–56^). The results, however, suggest that chronic stress has negative impact on these phenomena, although exceptions exist^57^. Furthermore, we lack evidence about specific implication of these phenomena in the expression of depression-like behaviors induced by stress protocols.

To our knowledge, we report here for the first time that a clear association exists between vulnerability to chronic stress, in terms of expression of depressive-like behavior, namely anhedonia, and occlusion of local DA-LTP in the mPFC. More precisely, a significant correlation was found between the level of sucrose preference and LTP magnitude in CMS rats. Interestingly, this result concerns a specific form of LTP which is dependent upon DA release, thus representing a point of convergence between reward-related behaviors and regulation of PFC connectivity. On the contrary, we did not detect any effect of CMS or vulnerability on DA-LTD: as the matter of fact, the available evidence suggests that DA-LTD is not linearly related to DA-LTP at the same pathway and the underlying expression mechanism is thought to be different^17^.

LTP compromission in our conditions can be related to several factors. In this work, we have shown that an increased AMPA/NMDA ratio of synaptic currents, which follows the reduction of sucrose preference in CMS rats, might underlie the compromission of LTP in vulnerable animals. This aspect is unlikely to indicate recent induction of LTP, since we did not observe rectification in the AMPA curve, that would be expected for calcium-permeable, GluA2-lacking receptors^30,58,59^. We did not find any other relevant result with these single-cell recordings. It is likely that the higher AMPA/NMDA ratio observed following loss of sucrose preference reflects deficient homeostatic or metaplastic processes, which might cause loss of plastic potential in extreme cases, *i.e.* when rats show anhedonia.

Regarding the metaplastic changes, we can argue that the threshold for synaptic potentiation is closely related to tonic DA release^11^, which is down-regulated in chronically stressed and depressive-like phenotype^6,7^. Nevertheless, by analyzing cFOS expression in neurons of DA producing regions, namely the VTA and SN, we were not able to detect any effect of stress in the activation of these areas.

Our results also show that if CMS animals are treated with acute ketamine, occlusion of DA-LTP is not detected, and both resilient and vulnerable animals display the expected final potentiation value which is generally obtained with these protocols on controls. Ketamine has been previously reported to alter DA-dependent synaptic plasticity in other synaptic pathways^60^. Such protective effect of ketamine on DA-LTP might be related to its action on both dopaminergic and glutamatergic transmission^23–25^. As for dopamine, ketamine was found to strongly promote VTA and SN activity in CMS rats. Although this result does not provide causal evidence of a dopaminergic role in ketamine’s action on DA-LTP, we can speculate that absence of DA-LTP occlusion with ketamine treatment might be related to VTA activity enhancement, as this is likely to increase background dopamine levels in the mPFC. Obviously, obtaining direct evidence supporting this idea, including measurements of dopamine neurons activity in ketamine-treated rats, both control and subjected to chronic stress, require future investigations. In addition, as it is the measure of choice in most CMS studies, we focused on anhedonia in our study, but evaluating other forms of depressive-like behavior would be very informative about the synaptic mechanisms investigated here.

Since mPFC is highly inter-connected to the other regions of the limbic system, any modification in PFC local circuitry is likely to affect its action on downstream targets. Among the others, amygdala is extremely important for producing behavioral responses to stress, as well as for the processing of stimuli emotional valence^44^. As the matter of fact, we found that synaptic drive from mPFC to BLA is reduced by CMS treatment, albeit this observation is not specific of vulnerable rats. This result seems stronger for the infralimbic portion of this pathway, so that the balance of activity between PL and IL populations of synapses is increased favoring the former one. Since it was previously observed that IL pathway exerts an inhibitory action on the BLA^43^, we can argue that CMS might lead to a reduction of this action that can contribute to the development of the depressive-like phenotype. Nevertheless, we did not evaluate here if there is any causal relationship between synaptic plasticity changes at local mPFC circuits and such unbalance of PL/IL -to-BLA synaptic drive, an insight that will require future investigations. Interestingly, we show here that ketamine seems to counteract this breakdown, thus suggesting a new circuit-specific action of this antidepressant treatment. These observations were obtained in vivo, at single synapse resolution and in a very deep nucleus, a result which is unprecedented and made possible by the use of the genetically-encoded sensor SynaptoZip^31^. Here, we demonstrate for the first time its applicability to investigate the activity of two different synaptic populations in the same animal, using different spectral versions, an approach which provides a ratiometric measure further reducing experimental variability.

## Conclusion

In conclusion, we have shown that chronic stress is able to produce dramatic effects on cortico-limbic circuitry, altering synaptic plasticity in the PFC in a manner which is dependent on the expression of the vulnerable phenotype, and reducing the synaptic drive of infralimbic PFC on the amygdala. The antidepressant ketamine was found to rescue both conditions, also involving the activation of DA-releasing regions that can contribute to the expression of the DA-dependent forms of synaptic plasticity investigated here. We believe that further investigation of these synaptic pathways can lead to a real comprehension of the neurophysiological mechanisms behind the expression of vulnerability and resilience to stress, as well as behind the antidepressant action of ketamine.

## Supplementary Information

Supplementary Information.

## Data Availability

The datasets generated during and/or analyzed during the current study are available from the corresponding author on reasonable request.
