# Supplementary material for: Occlusion of dopamine-dependent synaptic plasticity in the prefrontal cortex mediates the expression of depressive-like behavior and is modulated by ketamine
Source: Sci Rep. 2022 Jun 30;12:11055. doi: 10.1038/s41598-022-14694-w (PMC9246912; doi:10.1038/s41598-022-14694-w)
Supplement: Supplementary file 1 — Supplementary Information. [file 41598_2022_14694_MOESM1_ESM.pdf]

Supplementary information

Occlusion of dopamine-dependent synaptic plasticity in the prefrontal cortex mediates the expression of depressive-like behavior and is modulated by ketamine

Authors: Jacopo Lamanna[1,2*], Francesco Isotti[1£], Mattia Ferro[1,3£], Sara Spadini[1], Gabriella Racchetti[4], Laura Musazzi[5] & Antonio Malgaroli[1,2*]

Affiliations:
[1]Center for Behavioral Neuroscience and Communication (BNC), Vita-Salute San Raffaele University, 20132 Milan, Italy
[2]Faculty of Psychology, Vita-Salute San Raffaele University, 20132 Milan, Italy
[3]Department of Psychology, Sigmund Freud University, 20143 Milan, Italy
[4]Scientific Institute Ospedale San Raffaele, Division of Neuroscience, 20132 Milan, Italy
[5]School of Medicine and Surgery, University of Milano-Bicocca, 20900 Monza, Italy

[£] F.I. and M.F contributed equally to this work.

[*]**Co-corresponding authors:** lamanna.jacopo@hsr.it (J.L.) and malgaroli.antonio@unisr.it (A.M.) Center for Behavioral Neuroscience and Communication (BNC), Faculty of Psychology, Vita-Salute San Raffaele University, Via Olgettina 58, Milan 20132, Italy.

Supplementary methods

Animals. Only male rats were selected to minimize variability in stress response due to both sexual differences and/or estrous cycle. All efforts were made to minimize distress when not required by the protocols. Induction of anesthesia was always performed using sevoflurane (Abbvie, North Chicago, IL, USA).

Behavioral procedures. Swimming activity was extracted using the software analysis routine based on the extraction of an activity index; global thresholds were set to obtain three activity ranges: inactivity, moderate activity, and high activity. Inactivity reflected the absence of active movements except for those required to maintain the nose above water; high activity reflected struggling (trying to climb walls and moving actively with high strength in the water); moderate activity reflected lower swimming intensity or exploratory activity.

Acute slices preparation. After cutting, slices were submerged for 15 min in a recovery solution containing (in mM): 93 N-Methyl-D-glucamine (NMDG), 93 HCl, 2.5 KCl, 1.2 NaH_2PO_4, 30 $NaHCO_3$, 20 HEPES, 25 D-glucose, 5 sodium ascorbate, 2 Thiourea, 3 sodium pyruvate, 2 $MgSO_4$, 0.5 $CaCl_2$ (300 mOsm, adjusted to pH 7.4 with HCl)[1]. Slices were then transferred to an immersion chamber for the long recovery phase using the extracellular recording solution containing (in mM): 119 NaCl, 2.5 KCl, 1 NaH_2PO_4, 26.2 $NaHCO_3$, 2 $CaCl_2$, 1.3 $MgCl_2$, 11 D-glucose, bubbled with 95% O_2 and 5% CO_2 and warmed at 33.6 °C for 1 h. After recovery, slices were maintained at room temperature up to 6 h before being used for electrophysiological experiments.

Electrophysiological recordings. Voltage signals were amplified with an Axoclamp 2B amplifier (bridge mode, Axon Instruments, Foster City, CA, USA), then low-pass filtered (total gain = 1000; f_{LP} = 1 kHz) and digitalized at 20 kHz (16 bit) using the ITC-18 data acquisition interface (HEKA Elektronik, Lambrecht, Germany), controlled by a custom software developed in LabVIEW (National Instruments, USA). Current signals were filtered at 2–5 kHz and digitally acquired at 20 kHz using a 16-bit analog-to-digital interface (ITC-18; HEKA Elektronik, Lambrecht/Pfalz, Germany), controlled by custom acquisition software developed in LabVIEW (National Instruments, USA). Pyramidal cells in mPFC layer V were visually identified based on their shape using a CCD camera (SciCam Pro, Scientifica Ltd, UK) and IR illumination (with DIC optics or digital contrast) mounted on an upright microscope (Axioskop II FS Mot, Carl Zeiss, Jena, Germany). The stimulus intensity was always set to obtain about 50% of maximal linear response evaluated at the start of the

recording. After 10 min of stable recording at -70 mV, 20 EPSCs were collected at -70 mV and +40 mV holding potentials. Then, ACSF containing D-2-amino-phosphonovalerate (APV; 25 µM; Tocris Cookson, Bristol, UK) was perfused to block NMDA receptors thus obtaining the AMPA V-I curve at +40 mV, 0 mV and -70 mV holding potentials. EPSCs peak amplitudes for each condition were obtained using custom MATLAB (Mathworks) algorithms. NMDA currents at +40 mV were computed by subtracting the average AMPA response to each EPSC recorded in the absence of APV. Then, AMPA/NMDA ratio (at +40 mV) was obtained. Rectification index (RI) was computed as ($AMPA_{-70mV}$ / 70) / ($AMPA_{+40mV}$ / 40). For sake of comparability, AMPA response was normalized (dividing it by $AMPA_{-70mV}$) in the V-I plot and bar plot.

Image collection and analysis. For the automatic segmentation of synapses and quantification of normalized synaptic SB uptake. Briefly, normalized SB uptake was computed at the level of each individual synapse as (SB uptake – SB background) / (GZ expression – GZ background), with background measured in a region surrounding the bouton. For field-of-view (FOV)-averaged analysis, normalized synaptic SB uptake was averaged on the synapses of each FOV. In ratio analysis of SZ experiments, a ratio of average synaptic SB uptake measured at PL and IL synapses, respectively, was computed for each FOV. For the analysis of highly active synapses, the values of SB = (SB uptake – SB background) was compared to a threshold equal to median(SB)/2. For each FOV, the fraction of synapses with SB higher than this threshold were counted as highly active. A separate threshold was computed for experiments acquired with different microscopes (see above). For cFOS analysis, the same number of images were acquired at comparable locations of SN and VTA using low magnification images as reference; cells were automatically segmented on the NeuN channel using a custom watershed algorithm, then the average value of fluorescence for the cFOS channel was computed for each cell region. Then, a set of 20 circular regions with a diameter of 10.36 µm was randomly selected from each image in areas without NeuN+ cells, to obtain a background fluorescence distribution. A neuron was considered cFOS+ if its average fluorescence was higher than the mean + 2·SD of the background distribution. Then, percentage of cFOS+ cells in each field-of-view was computed as: 100 · (cFOS+ and NeuN+) / (NeuN+).

Statistical analysis. For the analysis of FOV-averaged synaptic SB uptake, a linear generalized mixed model (GLMM) was fitted using an inverse link function (assuming gamma-distributed data), random effects of subject and fixed effect of group; formula in

Wilkinsons's notation: y^{-1} ~ group + (1 | subject). Then, ANOVA was performed on the GLMM followed by analysis of all contrasts and B-H correction (for multiple groups) of the obtained p-values. For the analysis of fractions of highly active synapses, fractions in each FOV were fitted to a linear generalized mixed model (GLMM) assuming binomial distribution and logit link function, with number of synapses in each FOV as weight, random effects of subject, fixed effect of group and random interaction of group x microscope; formula: logit(y) ~ group + (1 | subject) + (1 | microscope:group); the random interaction group x microscope was not found significant). The ratio of the PL and IL binomial fractions was considered lognormally distributed[2] and fitted to a similar GLMM assuming normal distribution and logarithmic link function (formula: log(y) ~ group + (1 | subject) + (1 | microscope:group)). As above, ANOVA was performed on the GLMMs. For cFOS analysis, a GLMM was fitted to the fraction of cFOS-expressing cells assuming binomial distribution and logit link function, with number of NEUN+ cells in each FOV as weight, random effects of subject, fixed effect of group; formula: logit(y) ~ group + (1 | subject) (only FOVs with more than 10 cells and at least one cFOS+ were included); significance of group effect was then tested with a loglikelihood test; an observed power estimation on these GLMMs was performed using the *nmle* and *simr* packages of R (www.r-project.org) obtaining a power of >90%.

Supplementary figures

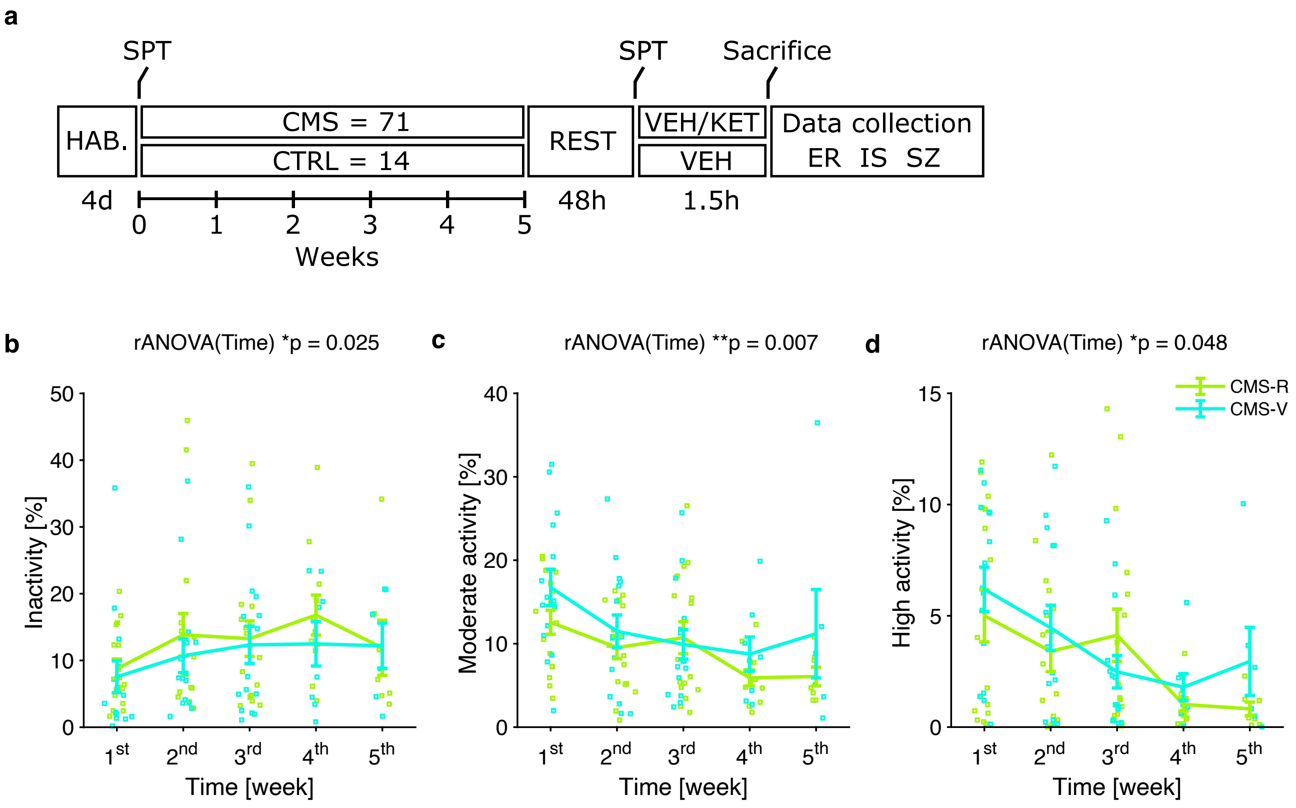

Supplementary Figure 1. a) Experimental design (HAB.: habituation; CTRL: controls; VEH: vehicle; KET: ketamine; ER: electrophysiological recordings; IS: immunostaining; SZ: SynaptoZip). **b-d)** Motivation for escape in the FST is reduced over weeks in both CMS-R and CMS-V rats, as shown by significant increase of inactivity (b; rANOVA: Time $F(4,44) = 3.826$, $p = 0.025$; Time x Group, $F(4,44) = 0.785$, n.s.) and parallel increase in moderate (c; rANOVA: Time $F(4,44) = 5.162$, $p = 0.007$; Time x Group, $F(4,44) = 0.781$, n.s.) and high (d; rANOVA: Time $F(4,44) = 3.449$, $p = 0.048$; Time x Group, $F(4,44) = 1.958$, n.s.) levels of swim activity.

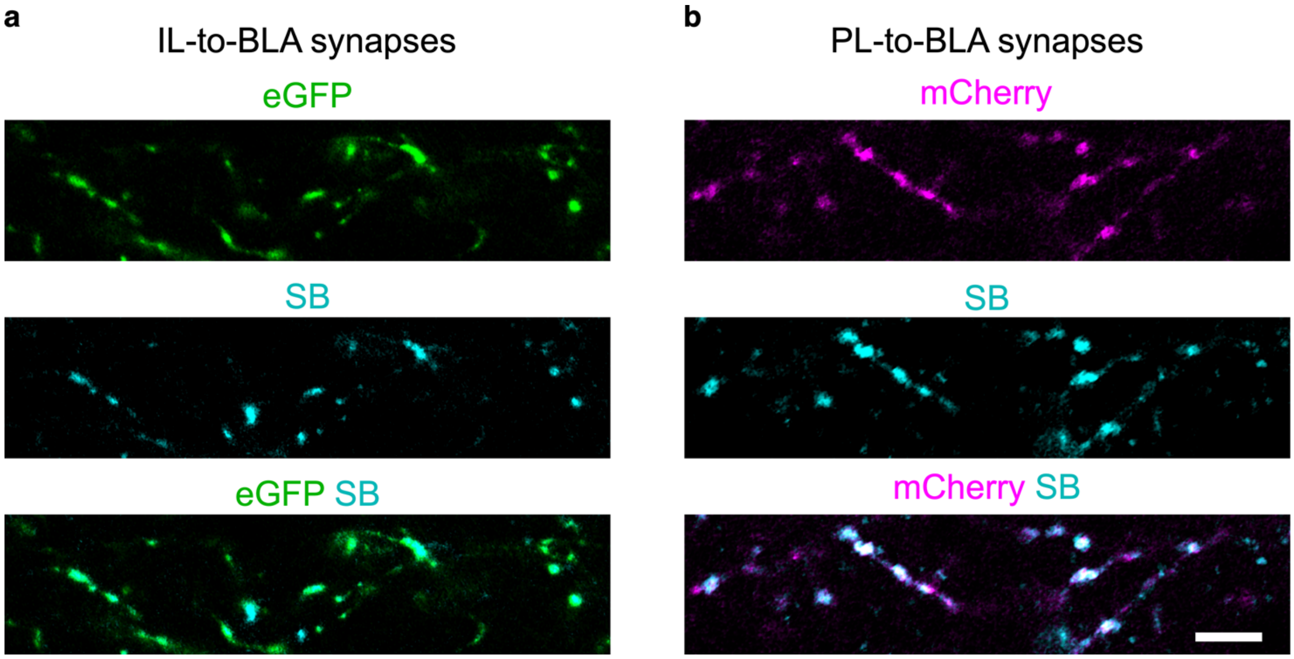

Supplementary Figure 2. eGFP-SZ and mCherry-SZ expression and SB uptake at individual presynaptic boutons in the basolateral amygdala (BLA). **a)** Top: eGFP-SZ (green) is expressed at individual synaptic boutons on axons coming from the infralimbic (IL) subregion of the medial prefrontal cortex (mPFC); Middle: SB (cyan) labels eGFP-SZ expressing synapses in awake, freely moving rats; Bottom: SB and eGFP show high colocalization (merge). **b)** At synaptic boutons on axons coming from the prelimbic (PL) mPFC, a similar pattern of expression is obtained for mCherry-SZ (magenta, top), as well as specific SB labelling (cyan, middle), with high colocalization (merge, bottom). Scale bar: 5 μm.

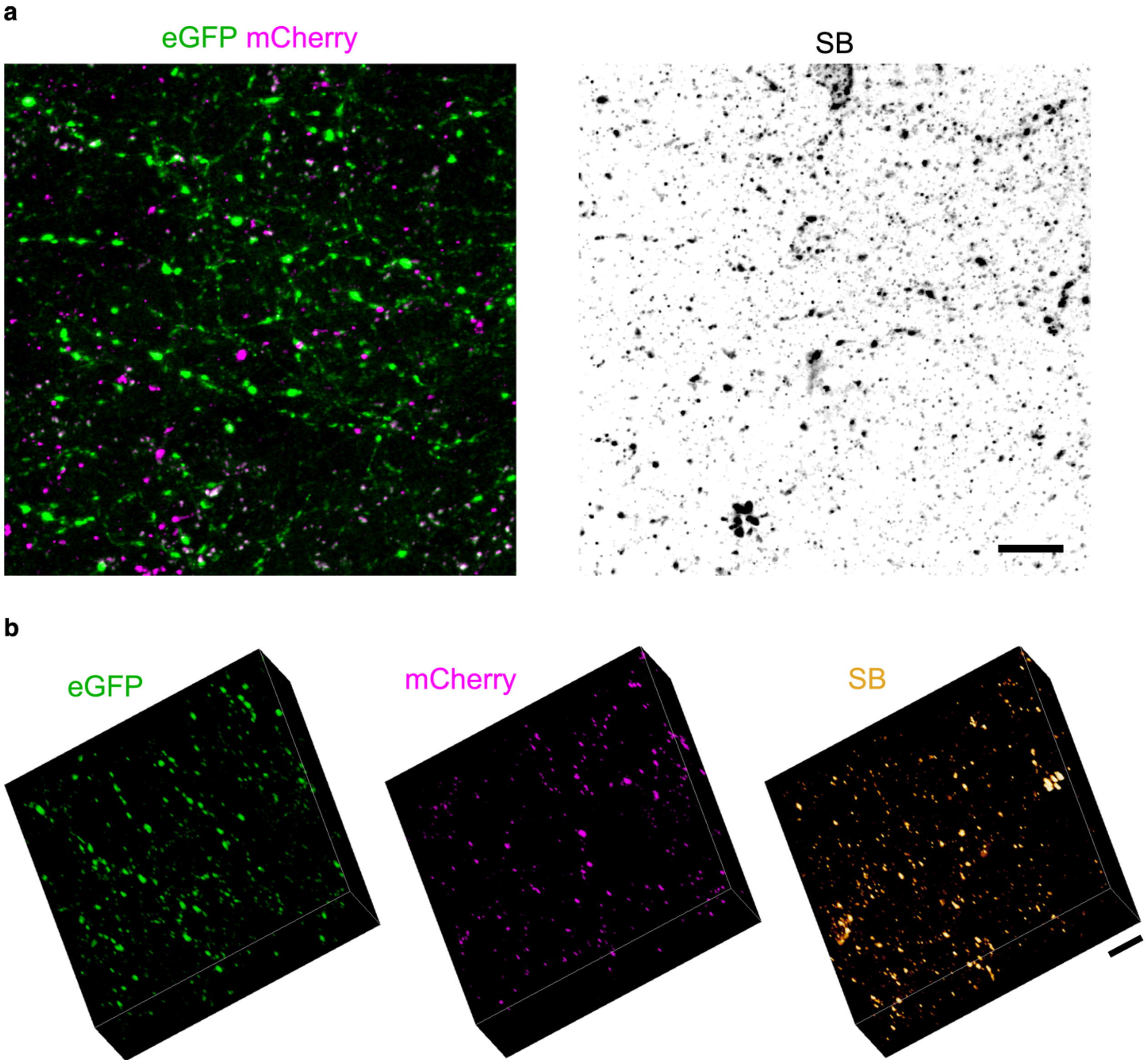

Supplementary Figure 3. SZ expression and SB uptake in the BLA. **a)** maximum intensity projection obtained from a confocal 3D stack acquired in the BLA. Left: synaptic boutons expressing either eGFP-SZ (green) or mCherry-SZ (magenta); right: SB signal (reverse gray LUT) in the same field. **b)** 3D view of the same stack shown in (a), with separated signals (SB shown with orange hot LUT). Scale bars: 10 μm (a,b).

REFERENCES

1. Ting, J. T. *et al.* Preparation of Acute Brain Slices Using an Optimized N-Methyl-D-glucamine Protective Recovery Method. *J. Vis. Exp.* 1–13 (2018). doi:10.3791/53825

2. Katz, D., Baptista, J., Azen, S. P. & Pike, M. C. Obtaining Confidence Intervals for the Risk Ratio in Cohort Studies. *Biometrics* **34**, 469 (1978).
